# Oral Health Management in Pediatric Surgical Inpatients: Development of Clinical Protocols Based on a Prospective Observational Study

**DOI:** 10.3390/dj14040201

**Published:** 2026-04-01

**Authors:** Claudia Capurro, Giulia Telini, Giulia Romanelli, Virginia Casali, Stefano Parodi, Nicola Laffi

**Affiliations:** 1 Department of Pediatric Dentistry and Orthodontics Unit, IRCCS Istituto Giannina Gaslini, 16147 Genoa, Italy; 2 Epidemiology and Biostatistics Unit, Scientific Directorate, IRCCS Istituto Giannina Gaslini, 16147 Genoa, Italy; stefanoparodi@gaslini.org

**Keywords:** child, hospitalization, surgery, pediatric surgery, inpatients, nutrition, oral health, oral hygiene, pediatric dentistry, preventive dentistry

## Abstract

**Background/Objectives:** Oral health is an essential component of general health, particularly in hospitalized pediatric patients undergoing surgery. Hospitalization may disrupt oral hygiene routines and dietary habits, increasing the risk of oral health deterioration. This prospective observational study aims to develop a standardized oral care protocol for pediatric patients hospitalized for surgical procedures by evaluating changes in oral health status, oral hygiene practices, and dietary habits between hospital admission and discharge. **Methods:** Children aged 0–17 years undergoing surgery and hospitalized for at least three nights were enrolled. Clinical oral examinations and caregiver-administered questionnaires were performed at admission and at discharge. Oral health status, plaque accumulation, gingival condition, oral pain, hygiene behaviors, and dietary habits were assessed. **Results:** In total, 118 patients were included. During hospitalization, plaque accumulation significantly increased and oral hygiene practices worsened. Dietary habits changed, with fewer daily meals and a slight reduction in cariogenic food and beverage intake. Oral hygiene instructions or dental examinations were documented in only 2.5% of patients. Based on these observations, a protocol was developed targeting hospitalized patients, their families, and healthcare staff, with the aim of improving oral health conditions during hospitalization. **Conclusions:** Pediatric surgical hospitalization is associated with a deterioration in oral hygiene behaviors and increased plaque accumulation. The implementation of standardized protocols and the dissemination of preventive oral health knowledge may transform hospitalization into an opportunity to improve oral health in children and adolescents.

## 1. Introduction

Oral health is increasingly recognized as a fundamental component of overall health, particularly in hospitalized patients undergoing surgical procedures. Poor oral hygiene and untreated oral diseases have been associated with several systemic conditions, including cardiovascular diseases, respiratory infections, diabetes mellitus, and adverse outcomes in medically compromised patients. The oral cavity can act as a reservoir for pathogenic microorganisms that may contribute to systemic inflammation and infection. Consequently, maintaining adequate oral hygiene is particularly important in vulnerable populations such as hospitalized patients, in whom systemic conditions, medications, and reduced self-care ability may further compromise oral health [1,2,3,4].

Furthermore, poor oral health increases the risk of postoperative infections, whereas perioperative oral care has been shown to reduce infectious complications and hospital stay across adult surgical populations [5,6,7].

During hospitalization, routine oral hygiene practices and dietary habits are often disrupted; this disruption may be more pronounced in pediatric patients, who depend largely on caregivers for daily oral care [8].

Systematic reviews and meta-analyses further support the role of non-pharmacological perioperative oral hygiene interventions in reducing postoperative pneumonia and surgical site infections, highlighting oral care as a low-cost and effective preventive strategy [6].

Despite growing evidence in adult populations, oral health management in hospitalized children, particularly those admitted to surgical wards, remains insufficiently explored. Pediatric patients present unique vulnerabilities related to dentition development, behavioral factors, and dietary habits, which may increase the risk of oral deterioration during hospital stays.

Previous reports have highlighted that oral and dental issues are frequently overlooked during general anesthesia and intensive care unit admissions in pediatric populations, even though these patients are at increased risk of dental trauma in intubation maneuvers, plaque accumulation, gingival inflammation, and oral discomfort [9].

Hospitalization and surgical care may significantly alter children’s oral hygiene routines and dietary patterns. Preoperative fasting, postoperative feeding modifications, use of medications, stress, and limited caregiver involvement can negatively affect the oral environment. Furthermore, attention in hospital settings is primarily focused on the underlying condition and perioperative management, while preventive oral care is often not systematically included in clinical protocols. This gap may lead to worsening oral conditions during hospitalization, potentially impacting comfort, nutrition, the quality of life and recovery [10].

The aim of the present study is to develop a standardized oral care protocol for pediatric patients hospitalized for surgical procedures at the Istituto di Ricerca e Cura a Carattere Scientifico (IRCCS) Giannina Gaslini pediatric hospital, based on the analysis of collected data on changes in oral health status, oral hygiene practices, and dietary habits between hospital admission and discharge, as well as on existing recommendations from the pediatric and general oral health prevention literature. The authors also intend to subsequently test and validate this protocol in a future study to assess its effectiveness and potential for implementation across other pediatric hospitals.

By addressing this underexplored area, the study seeks to contribute to improved awareness of oral health needs in hospitalized children and to promote the integration of preventive oral care into routine pediatric surgical care, outlining a structured oral care pathway to be applied in the pre-admission phase, throughout hospitalization, and after discharge.

## 2. Materials and Methods

### 2.1. Study Design and Setting

The present investigation is part of a larger prospective observational study conducted at the IRCCS Gaslini, Genoa, Italy, aimed at assessing changes in oral health status during hospitalization to develop ward-specific protocols for the improvement of oral health throughout the hospital stay.

The study protocol was reviewed and approved by the Regional Ethics Committee before the start of the investigation (CET-Liguria No. 294/2023-DB, ID 13233; approval date: 2 October 2023).

Financial support was provided by the Italian Ministry of Health through the 5x1000-2020 funding program (grant No. 5M-2020-23682532).

### 2.2. Participants

Children and adolescents consecutively admitted for surgical procedures under general anesthesia between 6 November 2023 and 30 April 2025 were assessed for eligibility. Patients aged between 0 and 17 years were considered eligible if their hospital stay lasted at least three consecutive nights.

Exclusion criteria included lack of cooperation, preventing oral examination and absence of a parent or legal guardian at the time of enrollment. Patients admitted to the oncology–hematology, bone marrow transplantation, cardiology, and pediatric cardiac surgery units were excluded from the present study, as their underlying systemic conditions significantly influence oral health status, and the extensive literature with established recommendations for the dental management of these patient groups is already available [11,12,13]. Their exclusion was also intended to ensure greater homogeneity of the study population.

Written informed consent was obtained from parents or legal guardians prior to enrollment. Age-appropriate assent was also obtained from the children whenever possible.

### 2.3. Baseline Assessment

Baseline data (T0) were collected within the first 24 h following hospital admission. Information on medical and dental history was obtained through a structured interview with caregivers and complemented by a review of electronic medical records (Galileo e-health.solutions NOEMALIFE©, Bologna, Italy). The variables included previous hospitalizations, the presence of chronic or acute systemic conditions, and ongoing pharmacological treatments, with particular attention to potentially cariogenic medication. The oral health–related information comprised daily oral hygiene practices, use of toothbrushes, toothpaste, or other oral care aids, history of previous dental treatments, and regular attendance at dental follow-up visits. The dietary habits were assessed by recording the number of daily meals, the types of foods and beverages consumed, and their potential cariogenicity. A comprehensive summary table of the collected variables is provided in the Supplementary Materials (Supplementary Table S1).

### 2.4. Clinical Oral Examination

Bedside oral examinations were carried out by three dental practitioners using standardized procedures. Prior to the start of the study, the examiners conducted preliminary joint assessments on a small number of patients to align their interpretation of the diagnostic criteria and standardize the examination procedures. The following indices were recorded: Dental caries experience was assessed using the Decayed, Missing, and Filled Teeth index for primary and permanent dentitions (dmft/DMFT) [14]. Gingival health was evaluated using the Modified Gingival Index (MGI), a non-invasive measure based on visual inspection of gingival color changes, swelling, erythema, hypertrophy, and bleeding, with scores ranging from 0 (healthy) to 4 (severe inflammation) [15]. Occlusal status was assessed using the Index of Orthodontic Treatment Need (IOTN), classifying malocclusions from grade 1 (no treatment required) to grade 5 (definite treatment need). In children younger than six years, the index was applied for screening purposes only [16]. Dental plaque accumulation was measured using the O’Leary Plaque Index (PI) [17]. After application of a plaque-disclosing agent, the percentage of tooth surfaces covered by plaque was calculated and categorized as low (0–25%), moderate (26–50%), high (51–75%), or very high (76–100%). Salivary pH was determined using colorimetric indicator strips in children who had refrained from eating or drinking for at least one hour prior to assessment.

### 2.5. Discharge Assessment

At hospital discharge (TD), caregivers completed a follow-up questionnaire documenting any new diagnoses, surgical procedures, or pharmacological treatments introduced during hospitalization.

Changes in dietary patterns (meal frequency and intake of cariogenic foods or beverages) and oral hygiene behaviors (performance, frequency, and type of oral care aids used) were recorded.

Additional items investigated whether dietary or oral hygiene counselling had been provided by hospital staff and whether a dental consultation had occurred during the hospital stay. A second oral examination was performed by three dental practitioners, using the identical indices and assessment procedures applied at T0. A comprehensive summary table of the collected variables is provided in the Supplementary Materials (Supplementary Table S2).

### 2.6. Statistical Analysis

Quantitative variables were described as means and standard deviations (sd) or medians and interquartile ranges (IQR), depending on the distribution (Gaussian or non-Gaussian). Normality was assessed by visual inspection of the related histograms. The Wilcoxon signed-rank test was used to assess changes in continuous variables. Categorical variables were described by absolute and relative (percentage) frequencies. Differences between T0 and TD were assessed by the McNemar test for binary variables and the Stuart–Maxwell homogeneity test for polychotomous ones [18]. A corresponding measure of effect size was obtained by the odds ratio (OR) for binary variables and by the cumulative OR for polytomous ordinal variables. The related estimates and the associated 95% confidence intervals (95%CI) were obtained by the conditional logistic regression model and by the cumulative logit model (with cluster-robust standard errors), respectively [19]. All statistical tests were two-tailed, and the threshold for statistical significance was set at *p* < 0.05. All analyses were carried out using the Stata/MP 18.0 statistical package (StataCorp LLC, College Station, TX, USA).

## 3. Results

**Figure 1:**
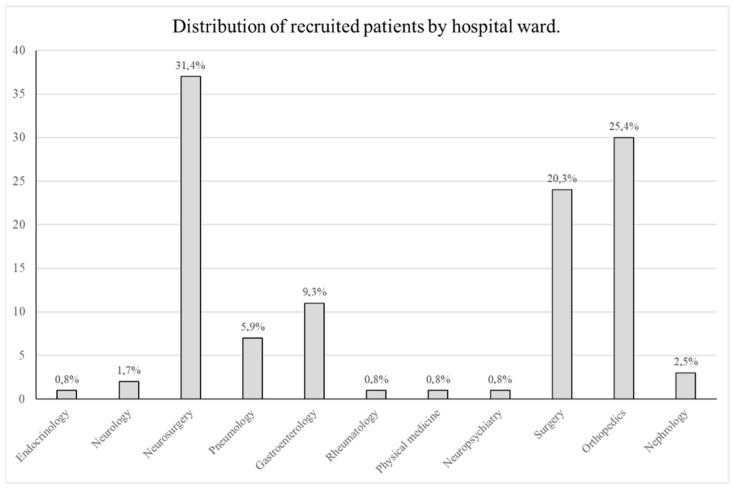
Distribution of recruited patients by hospital ward.

A total of 118 patients were enrolled in the study, all of whom underwent surgery under general anesthesia during their hospital stay. Figure 1 presents the originating hospital wards of the patients recruited for this study.

Children aged >12 years represented 35.6% of the population, followed by children aged 1–5 years (33.1%) and 6–11 years (29.7%), while infants under 1 year accounted for a small proportion (1.7%). Males slightly predominated over females (54.2% vs. 45.8%). Most patients (84.8%). presented a diagnosis of systemic disease at the time of admission.

Children receiving pharmacological therapy at home were 44.1% of the population, whereas in-hospital pharmacological treatment, excluding medication for general anesthesia, was administered to 82.2% of the cohort. Regarding hospitalization length, most patients were hospitalized for 4–15 days (67.8%), with shorter stays of 3 days observed in 17.0% of cases, and prolonged hospitalizations (>15 days) being relatively uncommon.

Table 1 summarizes the characteristics of the study population at T0.

A significant deterioration in hygiene practices was observed during hospitalization, with the proportion of patients not performing oral hygiene increasing from 8.5% at T0 to 34.8% at TD (*p* < 0.001). The use of oral care devices significantly declined during hospitalization, with non-use increasing from 0% at T0 to 23.2% at TD (*p* < 0.001). The frequency of toothbrush use decreased from 100% to 67.8% (*p* < 0.001).

The number of daily meals changed significantly over time (*p* < 0.001), with a marked shift toward fewer daily meals at discharge compared with admission.

Regarding diet quality, a modest but significant reduction in the consumption of cariogenic foods was observed at TD (92.0% vs. 83.9%; *p* = 0.020), along with a significant decrease in the intake of cariogenic beverages (67.0% vs. 53.6%; *p* = 0.002).

Table 2 describes changes in hygiene habits and dietary behaviors between T0 and TD.

Overall oral health status showed no statistically significant variation over time (*p* = 0.319), although a slight shift from good/very good to sufficient/fair categories was observed. The prevalence of oral pain decreased from 9.3% at T0 to 5.1% at TD, without reaching statistical significance (*p* = 0.225). Similarly, no significant differences were detected in PI distribution (*p* = 0.134), despite an increase in the proportion of patients with a very high PI at TD. However, when the original non-categorized variable was considered, the increase at TD was statistically significant (median 50.0, IQR 20.0–80.0, vs. 40.0, IQR 10.0–70.0, *p* = 0.001, T0-TD mean difference: −5.7, 95%CI: −10.0–−1.39). The MGI remained largely stable between the two time points (*p* = 0.215). Measurements of pH were available for 46 patients (39.0%). No statistically significant difference was observed between T0 (mean 6.5, sd 0.496) and TD (mean 6.3, sd 0.55, *p* = 0.164), corresponding T0-TD mean difference: 0.15, 95%CI: −0.03–0.33.

Table 3 reports changes in oral health–related outcomes between T0 and TD.

Table 4 shows dietary, oral care, and dental examination during patients’ hospitalization. Dietary recommendations were provided for 9.8% of patients. Oral hygiene instructions were provided exclusively during dental examinations, which were performed in only 2.5% of patients.

### 3.1. Proposed Oral Management Protocol for Pediatric Patients Undergoing Surgical Hospitalization at IRCCS Istituto Giannina Gaslini

Based on the findings of the present study and existing evidence on perioperative oral care, a structured protocol for the oral management of pediatric patients undergoing surgical hospitalization was developed. The protocol is designed to be simple, feasible, and easily integrated into routine pediatric surgical care. Some recommendations included in the proposed protocol, such as the use of chlorhexidine in specific clinical settings, should be interpreted within the context of local clinical policies, antimicrobial stewardship principles, and regulatory frameworks. These elements reflect a combination of available evidence and expert consensus and may therefore require adaptation according to local clinical practice and institutional guidelines. For clarity, the main operational components of the protocol are summarized in Table 5, while the following sections provide a concise description of the rationale and key elements of each phase.

### 3.2. Pre-Admission Phase

Whenever feasible, pediatric patients scheduled for elective surgical procedures should undergo a basic oral health assessment prior to hospital admission, either in a hospital-based setting or through referral to their primary dental care provider. The preoperative oral evaluation should include inspection of the oral cavity with specific attention to:

- Oral mucosa, noting the presence of lesions such as ulcers or aphthae, infectious foci (e.g., abscesses), or active infections (e.g., candidiasis or herpetic lesions).
- Gingival tissues, assessing signs of inflammation, including redness, edema, and bleeding.
- Teeth, identifying visible carious lesions suggestive of advanced disease.
- Oral hygiene status, by evaluating the presence of dental plaque, calculus, and gingivitis, with recommendation of professional oral hygiene if indicated.
- Orthodontic appliances, assessing their presence and the need for temporary removal prior to surgery.
- Teeth in exfoliation: identification of teeth in the exfoliation phase, assessment of the risk of accidental aspiration or ingestion during airway management and endotracheal intubation.
- Need for dental referral before the surgical intervention, based on clinical findings.

Caregivers will receive standardized information from recovery ward staff on the importance of maintaining optimal oral hygiene in the preoperative period, with the primary objective of reducing oral bacterial load in preparation for surgery, airway manipulation, and possible intubation. Recommendations should include:

- Toothbrushing at least twice daily, preferably after each meal, using an age-appropriated fluoridated toothpaste (first tooth–2 years: 1000 ppm fluoride, twice daily, grain-of-rice–sized amount; 2–6 years: 1000 ppm fluoride, twice daily, pea-sized amount; >6 years: 1450 ppm fluoride, twice daily, up to a full-length ribbon covering the toothbrush head).
- Use of chlorhexidine mouth rinses (0.12%), twice daily, starting 7 days before surgery and continuing for 7 days postoperatively, when age and cooperation allow, for oral cavity disinfection.
- Reminder to bring oral hygiene supplies and to adhere to the recommended oral care measures (oral hygiene and antiseptic rinses).
- Invitation to schedule a basic oral health assessment prior to hospital admission.

### 3.3. Hospitalization Phase

At admission, oral hygiene practices should be systematically assessed through a brief caregiver/children interview. All patients should have access to essential oral care devices (toothbrush, age-appropriate toothpaste and chlorhexidine mouth rinses), which should be provided by the hospital if not already available. Caregivers and, when appropriate, patients should receive brief oral hygiene instructions from trained healthcare staff, emphasizing the importance of continuing daily oral care throughout hospitalization, including the perioperative period.

Oral hygiene should be actively encouraged by hospital staff at least once daily during the hospital stay, considering postoperative conditions, pain, and patient comfort. Daily inspection of the oral cavity to identify potential deterioration in oral hygiene or mucosal conditions should be integrated into routine ward medical rounds.

In cases of prolonged hospitalization, poor baseline oral health, or inability to perform adequate oral hygiene independently, a dental consultation should be considered.

During hospital stay, particular attention should be given to the prevention and management of postoperative nausea and vomiting (PONV), which is a common complication in pediatric surgical patients. Adequate control of PONV is essential to improve patient comfort and to reduce the risk of dehydration, delayed oral intake, and potential adverse effects on oral health. Episodes of vomiting may expose the oral cavity to gastric acids, increasing the risk of dental erosion and mucosal irritation. For this reason, oral hygiene measures should be temporarily adapted following emetic episodes, including gentle rinsing of the mouth with water and bicarbonate solutions to help neutralize oral acidity and postponement of toothbrushing for an appropriate period (approximately 30–60 min) to avoid enamel damage.

For patients admitted to intensive care units or postoperative recovery settings where caregiver access is limited, oral hygiene should be performed at least once daily by trained healthcare staff using appropriate devices, such as gauze or oral sponges soaked in chlorhexidine solution (0.20%).

During hospital stay, a non-cariogenic dietary regimen should be adopted. Dietary habits should be actively monitored by hospital staff, with particular attention to discouraging frequent snacking and the consumption of cariogenic foods and beverages, especially outside regular mealtimes. The availability of sticky sweets, sugar-containing beverages, and sugary snacks between meals should be avoided. In addition, hospitals should prepare and disseminate educational brochures for caregivers and patients, providing clear and accessible information on the cariogenic potential of foods and beverages and promoting healthier dietary choices.

During hospitalization, specific information should also be provided regarding medications that may negatively affect oral health. Attention should be given to drugs associated with xerostomia, such as anticholinergics, antihistamines, opioids, and certain antiemetics, as well as to acidic or sugar-containing formulations, including syrups and liquid medications commonly prescribed in pediatric patients. Caregivers and patients should receive appropriate guidance on oral cavity care, including reinforced oral hygiene measures, the use of saliva substitutes when indicated, and mouth rinses with bicarbonate solutions to help neutralize oral acidity.

### 3.4. Discharge Phase

At discharge, caregivers should receive oral hygiene and dietary counseling, including recommendations to resume regular oral hygiene routines, to schedule routine dental follow-up visits, to avoid the consumption of cariogenic foods and beverages, and to limit frequent snacking between meals.

Each patient should also receive tailored information regarding the type of medications prescribed for home use, with particular attention to their potential impact on oral health.

For patients who experienced significant deterioration in oral hygiene practices or increased plaque accumulation during hospitalization, referral to pediatric dental services should be recommended.

Written educational materials may be provided to support adherence after discharge.

### 3.5. Healthcare Staff Training

A structured and mandatory training program should be implemented for healthcare staff involved in the perioperative management of pediatric patients to ensure consistent and appropriate application of the protocol. Training activities should aim to strengthen competence in pediatric oral health prevention, early risk recognition, and interdisciplinary collaboration within the hospital setting.

Training programs should include the following elements:

- Educational sessions organized and delivered by pediatric dentists or dental professionals with specific expertise in pediatric oral health.
- Regularly scheduled training activities, with updates provided at least on an annual basis to incorporate emerging evidence.
- Promotion of interdisciplinary collaboration between dental professionals, surgeons, anesthesiologists, and nursing staff.
- Support for early identification of oral health–related risk factors, implementation of preventive measures, and timely referral to pediatric dental services.
- Periodic evaluation of training effectiveness through internal audits or quality indicators to monitor adherence to the protocol and identify areas for improvement.

This proposed protocol aims to facilitate the integration of preventive oral health measures into pediatric surgical care pathways, addressing the gaps in oral hygiene practices and counseling identified during hospitalization. Future interventional studies are warranted to evaluate the feasibility, effectiveness, and clinical impact of this protocol on oral health outcomes and postoperative recovery in pediatric surgical populations.

## 4. Discussion

The present prospective observational study investigated changes in oral hygiene behaviors, dietary habits and oral health status in pediatric patients undergoing surgical hospitalization, comparing findings at admission and discharge. Overall, the results indicate that surgical hospitalization is associated with a deterioration in oral hygiene practices and increased plaque accumulation, despite relatively stable clinical oral health indices and an apparent improvement in dietary behaviors.

Regarding clinical oral health outcomes, no statistically significant differences were observed between admission and discharge in overall oral health status, gingival condition, or oral pain. These findings may be explained by the relatively short duration of hospitalization for most patients, which may be insufficient to induce measurable changes in caries experience or overt gingival inflammation. However, when plaque accumulation was analyzed as a continuous variable rather than categorized classes, a statistically significant increase was detected at discharge. This finding is clinically meaningful, as dental plaque represents an early and sensitive indicator of oral hygiene disruption and a key risk factor for the development of gingivitis and dental caries in pediatric [20,21].

In contrast to the relative stability of clinical indices, oral hygiene behaviors significantly worsened during hospitalization. Both the frequency of oral hygiene practices and the use of oral care devices declined markedly between admission and discharge, with more than one-third of patients not performing any oral hygiene by the end of the hospital stay. These findings are consistent with previous reports showing that oral care is frequently neglected in hospitalized children, particularly when attention is focused on acute medical or surgical management [8,10,22]. In pediatric patients, this issue may be further exacerbated by postoperative discomfort, fatigue, altered daily routines, and strong dependence on caregivers for daily oral hygiene assistance. Given the observational design of the study, the associations observed between hospitalization and changes in oral hygiene behaviors and plaque accumulation should be interpreted with caution, as they may be influenced by several confounding factors such as underlying systemic conditions, pharmacological treatments, and baseline clinical status.

Interestingly, dietary behaviors showed an opposite trend. During hospitalization, children exhibited a significant reduction in the number of daily meals and a modest but statistically significant decrease in the consumption of cariogenic foods and beverages. This pattern is likely attributable to hospital dietary regulations, perioperative fasting protocols, and structured meal schedules, which may temporarily limit access to sugary snacks and drinks. Similar associations between structured dietary environments and improved diet quality have been described in pediatric populations [23]. The observed reduction in meal intake should also be interpreted considering the children’s likely discomfort and general malaise during hospitalization, which could have contributed to a decreased appetite. Although these changes may exert a short-term protective effect against caries development, they appear insufficient to offset the negative impact of impaired oral hygiene on plaque accumulation.

A particularly concerning finding of the present study is the extremely limited provision of oral and nutritional counseling during hospitalization. Only a very small proportion of patients received oral hygiene instructions from hospital staff, and fewer than one in ten were provided with dietary recommendations. Dental examinations were also rarely documented. Previous studies have highlighted that hospitalized children often present unmet oral health needs and face multiple barriers to receiving adequate dental care during inpatient stays [8,10,22]. Moreover, healthcare professionals frequently report limited training, time constraints, and a lack of standardized protocols as major obstacles to delivering effective oral care in pediatric hospital settings [24].

Taken together, these findings highlight a substantial gap in comprehensive pediatric surgical care. While evidence from adult surgical populations suggests that perioperative oral care interventions can reduce postoperative complications and improve clinical outcomes, pediatric-specific data remain scarce. Nevertheless, the integration of simple, preventive oral health measures, such as caregiver education, routine oral hygiene delivery or instructions, and the availability of basic oral care devices, may represent a low-cost and feasible strategy to preserve and improve oral health in hospitalized children, as supported by current pediatric oral health literature [25], and directly informed the development of the proposed clinical protocol.

The significant decline in oral hygiene practices observed during hospitalization supports the inclusion of structured daily oral hygiene encouragement and systematic assessment of oral hygiene at admission. Similarly, the increase in plaque accumulation, even in the absence of short-term clinical deterioration, highlights the importance of maintaining regular oral hygiene routines during the hospital stay. The very limited provision of oral hygiene instructions and dental consultations documented in our cohort further justifies the integration of standardized caregiver education, availability of oral hygiene devices, and the possibility of dental referral within the hospitalization phase of the protocol. Moreover, the observed changes in dietary patterns during hospitalization further highlight the potential role of structured dietary monitoring and guidance in supporting healthier dietary behaviors and limiting exposure to cariogenic foods and beverages. For these reasons, the protocol includes specific recommendations addressing dietary counseling and the promotion of non-cariogenic dietary habits during the hospital stay. Taken together, the empirical findings of this study highlight modifiable factors that can be addressed through simple preventive interventions, which are reflected in the operational components of the proposed clinical protocol.

The main strength of this study lies in its prospective design and the use of standardized clinical oral examinations, which allowed for a systematic evaluation of changes in oral health and related behaviors during hospitalization. Additionally, the study addresses a largely underexplored area in pediatric surgical care and translates observational findings into a structured oral health management protocol. The proposed protocol should not be interpreted as being entirely derived from the findings of the present observational study. Rather, it represents a framework partially informed by the results of this cohort and partially grounded in existing pediatric dentistry literature and clinical preventive strategies. Some recommendations included in the protocol, such as the use of chlorhexidine in specific clinical situations, should be interpreted within the context of local clinical policies, antimicrobial stewardship principles, and regulatory frameworks, and may therefore require adaptation across different healthcare settings.

This study has several limitations that should be considered when interpreting the findings. First, the study was conducted in a single tertiary pediatric hospital, which may limit the generalizability of the results to other healthcare settings with different organizational structures, resources, or patient populations. Second, the study population was heterogeneous with respect to underlying systemic diseases and pharmacological treatments, which may influence oral health status and hygiene behaviors. Third, part of the information on oral hygiene practices and dietary habits was obtained through caregiver-reported questionnaires, which may be subject to recall bias, social desirability bias, and potential Hawthorne effects. In addition, the study did not include long-term follow-up, preventing evaluation of whether the short-term increase in plaque accumulation observed during hospitalization may translate into clinically significant caries or periodontal outcomes over time. Finally, although examiners followed a standardized examination protocol and performed preliminary joint assessments to harmonize diagnostic criteria, a formal statistical evaluation of inter-examiner agreement was not conducted.

## 5. Conclusions

This study highlights that pediatric surgical hospitalization may negatively affect oral hygiene behaviors, leading to increased plaque accumulation even in the absence of short-term changes in clinical oral health indices. While hospital dietary regimens appeared to promote less cariogenic eating patterns, they are not used as an opportunity to educate children and their families to correct eating habits.

The minimal involvement of healthcare staff in providing oral hygiene and dietary guidance underscores the need to better integrate preventive oral health measures into pediatric surgical care.

Future controlled studies are needed to validate the proposed protocol and to determine whether its implementation results in measurable improvements in oral health indicators, such as reductions in plaque index and improvements in oral hygiene practices among hospitalized pediatric patients. Such studies should evaluate adherence to the protocol and assess relevant outcomes, including changes in plaque index, rates of documented oral hygiene counseling, and other indicators of preventive oral care during hospitalization.

## Figures and Tables

**Table 1:** Patient characteristics at T0.

Patient Characteristics	N	%
Age (years)		
<1	2	1.7
1–5	39	33.1
6–11	35	29.7
≥12	42	35.6
Gender		
Males	64	54.2
Females	54	45.8
Diagnosis of systemic disease		
No	18	15.3
Yes	100	84.8
Baseline pharmacologic therapy		
No	66	55.9
Yes	52	44.1
In-hospital pharmacologic therapy *		
No	21	17.8
Yes	97	82.2
Length of hospitalization (days)		
3	20	17.0
4–15	80	67.8
16–30	13	11.0
>30	5	4.2

* Excluding medication for general anesthesia.

**Table 2:** Changes in hygiene habits and dietary behaviors between T0 and TD.

	T0		TD				
Patient Characteristics	N	%	N	%	OR	95%CI	*p*
Hygiene practices (N = 118)							<0.001
No	10	8.5	41	34.8	ref	-	
Yes	108	91.5	77	65.2	0.17 ^#^	0.09–0.33	
Oral care devices (N = 108) *							<0.001
No	0	0.0	25	23.2	ref	-	
Yes	108	100.0	83	76.2	n.e.	n.e.	
Number of daily meals (N = 112) **							<0.001
<3	4	3.5	5	4.5			
3	11	9.8	43	38.4			
4	31	27.7	31	27.7			
5	47	42.0	21	18.8			
>5	19	17.0	12	10.7	0.32 ^##^	0.21–0.49	
Cariogenic food (N = 112) **							0.020
No	9	8.0	18	16.1	ref	-	
Yes	103	92.0	94	83.9	0.25 ^#^	0.07–0.89	
Cariogenic beverages (N = 112) **							0.002
No	37	33.0	52	46.4	ref	-	
Yes	75	67.0	60	53.6	0.21 ^#^	0.07–0.62	

*p* = *p*-value obtained by the Stuart–Maxwell test for paired samples. * 10 missing values due to patients not performing any oral hygiene procedure. ** 6 missing values due to patients receiving enteral or parenteral nutrition via percutaneous endoscopic gastrostomy (PEG). OR = Odds Ratio; ^#^ Estimate from conditional logistic regression; ^##^ Cumulative odds ratio estimates from ordinal logistic regression; n.e. = not evaluable due to zero events observed in one cell; ref = reference.

**Table 3:** Clinical oral examination at T0 and TD.

	T0		TD				
Patient Characteristics	N	%	N	%	OR	95%CI	*p*
Oral health status (N = 118)							0.319
Poor	21	17.8	21	17.8			
Sufficient/Fair	39	33.0	43	36.4			
Good/Very good	58	49.2	54	45.8	0.91 ^##^	0.76–1.1	
Oral pain (N = 118)							0.225
No	107	90.7	112	94.9	ref	-	
Yes	11	9.3	6	5.1	0.54 ^#^	0.20–1.5	
PI (N = 104) *							0.134
Low	39	37.5	35	33.7			
Moderate	30	28.9	27	26.0			
High	11	10.6	10	9.6			
Very high	24	23.1	32	30.8	1.3 ^##^	1.0–1.7	
MGI (N = 118)							0.215
0	29	24.6	25	21.2			
1	47	39.8	46	39.0			
2	27	22.9	31	26.3			
3	11	9.3	12	10.2			
4	4	3.4	4	3.4	1.2 ^##^	1.0–1.4	

*p* = *p*-value obtained by the Stuart–Maxwell test for paired samples. * 14 missing values due to patients not allowing the use of a plaque disclosing agent. OR = Odds Ratio; ^#^ Estimate from conditional logistic regression; ^##^ Cumulative odds ratio estimates from ordinal logistic regression.

**Table 4:** Oral and Nutritional Patient Care.

	N	%
Dietary recommendations (N = 112) *		
No	101	90.2
Yes	11	9.8
Oral hygiene instructions (N = 118)		
No	115	97.5
Yes	3	2.5
Dental exam (N = 118)		
No	115	97.5
Yes	3	2.5

* 6 missing values due to patients receiving enteral or parenteral nutrition via percutaneous endoscopic gastrostomy (PEG).

**Table 5:** Summary of recommendations of the IRCCS Istituto Giannina Gaslini Oral Management Protocol for Pediatric Patients Undergoing Surgical Hospitalization.

Phase	Domain	Key Recommendations
Pre-admission	Oral assessment	Preoperative evaluation of oral mucosa, gingiva, teeth, oral hygiene status, orthodontic appliances, dentition stage, and exfoliating teeth.
	Risk identification	Identification of oral infections, caries, gingivitis, poor oral hygiene, and aspiration risk.
	Dental referral	Referral to dental services when clinically indicated.
	Caregiver education	Standardized information on the importance of preoperative oral hygiene.
	Oral hygiene measures	Twice-daily toothbrushing with age-appropriate fluoridated toothpaste; use of chlorhexidine mouth rinses when feasible.
Hospitalization	Oral hygiene assessment	Assessment of oral hygiene practices at admission and provision of oral care devices.
	Daily oral care	Daily encouragement of oral hygiene and oral cavity inspection during ward rounds.
	Dental consultation	Consideration of dental consultation in prolonged hospitalization or poor oral health.
	PONV * management	Adaptation of oral hygiene after vomiting episodes, including oral rinsing and delayed toothbrushing.
	Intensive care settings	Staff-assisted oral care in intensive care units using appropriate devices.
	Diet	Adoption of a non-cariogenic hospital diet and monitoring of dietary habits.
	Medications	Guidance on drugs associated with xerostomia and acidic or sugar-containing formulations.
Discharge	Counseling	Oral hygiene and dietary counseling at discharge.
	Medications	Tailored information on home medications and their potential oral health impact.
	Dental follow-up	Recommendation of routine dental follow-up visits.
	Referral	Referral to pediatric dental services when oral health deterioration occurs.
Healthcare staff training	Training program	Structured and mandatory training programs led by pediatric dental professionals.
	Updates and evaluation	Regular updates (at least annually) and evaluation through audits or quality indicators.

* postoperative nausea and vomiting (PONV).

## Data Availability

The datasets presented in this article are not readily available because they contain sensitive patient information and are subject to institutional data protection regulations. Requests to access the datasets should be directed to the corresponding author.

## Supplementary Materials

The following supporting information can be downloaded at https://www.mdpi.com/article/10.3390/dj14040201/s1, Table S1: Structure of the T0 Data Collection Instrument; Table S2: Structure of the TD (Discharge) Data Collection Instrument.

## Abbreviations

The following abbreviations are used in this manuscript:

DMFT	Decayed, Missing, and Filled Teeth index, permanent dentition
dmft	Decayed, Missing, and Filled Teeth index, primary dentition.
IOTN	Index of Orthodontic Treatment Need
IRCCS	Istituto di Ricovero e Cura a Carattere Scientifico
MGI	Modified Gingival Index
PEG	Percutaneous Endoscopic Gastrostomy
PI	Plaque Index
PONV	Postoperative Nausea and Vomiting
sd	Standard Deviations
T0	Time of Admission
TD	Time of Discharge
