# Supplementary material for: Oral Health Management in Pediatric Surgical Inpatients: Development of Clinical Protocols Based on a Prospective Observational Study
Source: Dent J (Basel). 2026 Apr 1;14(4):201. doi: 10.3390/dj14040201 (PMC13115171; doi:10.3390/dj14040201)
Supplement: Supplementary file 1 [file dentistry-14-00201-s001.zip › Supplementary Table S1.pdf]

Supplementary Table S1. Structure of the T0 Data Collection Instrument

Domain	Subdomain	Variables Collected
**1. Demographic and Hospitalization Data**	Patient demographics	Sex, age, weight, country of birth
	Hospitalization details	Date of admission, hospital ward, reason for hospitalization, ICD code
**2. Family and Socio-Demographic Background**	Caregiver characteristics	Primary caregiver
	Parental background	Country of birth (mother/father), educational level, employment status
	Household structure	Number of siblings, adoption/foster care status, residence in community facilities
**3. Medical History**	Perinatal history	Gestational age
	Systemic condition	Main systemic diagnosis (ICD), year of diagnosis
	Comorbidities	Associated medical conditions
	Previous healthcare utilization	Previous hospitalizations, previous surgical procedures
**4. Pharmacological Therapy**	Current medications	Active principles, formulation, daily frequency, duration
	Oral health-related effects	Reported oral side effects, oral health precautions
	Previous therapies	Discontinued pharmacological treatments, ongoing rehabilitative therapies
**5. Dietary Habits**	Early feeding history	Type, modality, and duration of breastfeeding
	Current feeding modality	Oral, enteral, or mixed feeding
	Daily dietary pattern	Number of meals per day
	Beverage consumption	Water, milk, fruit juices, soft drinks, tea/herbal drinks
	Cariogenic food intake	Sugar, honey, candies, baked sweets, chocolate, others

Domain	Subdomain	Variables Collected
**6. Oral Hygiene Practices**	Hygiene performance	Frequency and daily distribution of oral hygiene practices
	Hygiene aids used	Manual/electric toothbrush, fluoride/non-fluoride toothpaste, mouthrinses, gels, interdental aids
	Supervision	Performer of oral hygiene (child/caregiver)
	Preventive awareness	Knowledge of fluoride use
**7. Dental History**	Dental attendance	Previous dental visits
	Oral symptoms	Presence and type of oral pain
	Orthodontic history	Current or previous orthodontic treatment
	Oral habits	Oral breathing, digit sucking, pacifier use, onychophagia, others
	Sleep-related conditions	Snoring, sleep apnea
**8. Intraoral Examination**	Dentition status	Type of dentition, number of deciduous/permanent teeth
	Caries indices	dmft/DMFT
	Periodontal indices	Modified Gingival Index (MGI), Plaque Index (PI)
	Dental anomalies	Structural, numerical, positional, or chromatic alterations
	Oral mucosa	Color, integrity, hydration status, presence of lesions
	Other findings	Erosions, abrasions, abscesses, fistulae
**9. Extraoral Examination**	Functional assessment	Mouth opening pattern and range
	Pain assessment	Presence of articular or muscular pain
**10. Salivary Assessment**	Salivary parameters	Unstimulated salivary flow rate, buffering capacity (pH)
