# Supplementary material for: Oral Health Management in Pediatric Surgical Inpatients: Development of Clinical Protocols Based on a Prospective Observational Study
Source: Dent J (Basel). 2026 Apr 1;14(4):201. doi: 10.3390/dj14040201 (PMC13115171; doi:10.3390/dj14040201)
Supplement: Supplementary file 1 [file dentistry-14-00201-s001.zip › Supplementary Table S2.pdf]

Supplementary Table S2. Structure of the TD (Discharge) Data Collection Instrument

Domain	Subdomain	Variables Collected
**1. Hospitalization Overview**	Admission and discharge data	Ward, admission date, discharge date, transfer status
	Follow-up timepoints	Dates of T0, intermediate assessments (T1–T>3), and TD
	Anthropometrics	Weight at discharge
**2. Family Context During Hospitalization**	Caregiver presence	Caregiver(s) assisting the child during hospitalization (mother, father, healthcare staff, other)
**3. Medical Course During Hospitalization**	New diagnoses	Pathologies diagnosed during hospitalization
	Interventions	Surgical procedures performed during hospitalization
	Therapies	Pharmacological or medical therapies introduced during hospitalization
**4. Dietary Habits During Hospitalization**	Dietary modifications	Changes in diet type, quantity, or type of liquids consumed
	Feeding modality	Oral, enteral, or mixed feeding
	Meal frequency	Number of meals per 24 hours
	Beverage consumption	Water, milk, fruit juices, soft drinks, tea/herbal drinks
	Cariogenic food intake	Sugar, honey, candies, baked sweets, chocolate, others
	Nutritional counseling	Dietary advice provided during hospitalization and at discharge
**5. Oral Hygiene Practices During Hospitalization**	Hygiene performance	Execution of oral hygiene maneuvers
	Habit changes	Variations in oral hygiene habits during hospitalization
	Hygiene aids used	Toothbrush (manual/electric), toothpaste (fluoridated/non-fluoridated), mouthrinses, gels, interdental aids
	Frequency and timing	Daily frequency and distribution of oral hygiene practices

Domain	Subdomain	Variables Collected
	Supervision	Performer of oral hygiene maneuvers (child/caregiver)
	Preventive counseling	Oral hygiene instructions provided during hospitalization and at discharge
**6. Dental Events During Hospitalization**	Dental consultation	Dental visits performed during hospitalization
	Oral symptoms	Onset and type of oral pain
	Oral habits	Changes in parafunctional habits
	Sleep-related variables	Snoring, sleep apnea, polysomnography, CPAP use
	Salivary changes	Subjective variation in salivary flow
**7. Intraoral Examination at Discharge**	Global oral health status	Overall oral health assessment
	Therapy-related manifestations	Oral manifestations related to systemic disease or therapies
	Dentition status	Type of dentition, number of deciduous/permanent teeth
	Caries indices	dmft/DMFT
	Periodontal indices	Modified Gingival Index (MGI), Plaque Index (PI)
	Dental anomalies	Structural, numerical, positional, or chromatic alterations
	Oral mucosa	Color, integrity, hydration, presence of lesions
	Other findings	Erosions, abrasions, abscesses, fistulae
**8. Extraoral Examination**	Functional assessment	Mouth opening pattern and range
	Pain assessment	Presence of articular or muscular pain
**9. Salivary Assessment**	Salivary parameters	Unstimulated salivary flow rate, buffering capacity (pH)
